# TRAINING THE NEXT GENERATION OF GERONTOLOGICAL LEADERSHIP: WHO AND HOW?

**DOI:** 10.1093/geroni/igac059.1339

**Published:** 2022-12-20

**Authors:** Neil Charness, Patricia Heyn, James Appleby

## Abstract

Knowing how to train the next generation of gerontological leaders involves understanding where we are now and where we want to be in the coming decades. We outline the results of a survey of the membership of Directors of Aging Centers. The Directors of Aging Centers interest group in GSA has representation from the USA, Canada, and Europe. A survey was sent to the membership in late December with reminders in January and had 31 respondents. We discuss the results of the survey, highlighting the demographics of the current leadership (Neil Charness), perceived need for training by current leaders (Peter Lichtenberg), and consensus content of leadership training programs (Patricia Heyn). Patricia D’Antonio provides a perspective on GSA’s approach to professional development programs and avenues for soliciting funding for leadership training. Our discussant (James Appleby, CEO of GSA) will reflect on the need for training in the context of building the next generation of gerontological leadership. We plan to devote significant symposium time to solicit audience input on next steps for supporting the effort to improve the quantity, quality, and diversity of the gerontological leadership workforce.

